# Efficient In Vitro and In Vivo Activity of Glyco-Engineered Plant-Produced Rabies Monoclonal Antibodies E559 and 62-71-3

**DOI:** 10.1371/journal.pone.0159313

**Published:** 2016-07-18

**Authors:** Tsepo Lebiletsa Tsekoa, Therese Lotter-Stark, Sindisiwe Buthelezi, Ereck Chakauya, Stoyan H. Stoychev, Claude Sabeta, Wonderful Shumba, Baby Phahladira, Steve Hume, Josh Morton, Charles E. Rupprecht, Herta Steinkellner, Michael Pauly, Larry Zeitlin, Kevin Whaley, Rachel Chikwamba

**Affiliations:** 1 Biosciences Unit, Council for Scientific and Industrial Research, Pretoria, South Africa; 2 ARC-Onderstepoort Veterinary Institute, Onderstepoort, South Africa; 3 Mapp Biopharmaceutical, San Diego, California, United States; 4 Kentucky Bioprocessing, Owensboro, Kentucky, United States; 5 The Wistar Institute, Philadelphia, Pennsylvania, United States; 6 Department of Applied Genetics and Cell Biology, University of Natural Resources and Life Sciences, Vienna, Austria; The Scripps Research Institute and Sorrento Therapeutics, Inc., UNITED STATES

## Abstract

Rabies is a neglected zoonotic disease that has no effective treatment after onset of illness. However the disease can be prevented effectively by prompt administration of post exposure prophylaxis which includes administration of passive immunizing antibodies (Rabies Immune Globulin, RIG). Currently, human RIG suffers from many restrictions including limited availability, batch-to batch inconsistencies and potential for contamination with blood-borne pathogens. Anti-rabies monoclonal antibodies (mAbs) have been identified as a promising alternative to RIG. Here, we applied a plant-based transient expression system to achieve rapid, high level production and efficacy of the two highly potent anti-rabies mAbs E559 and 62-71-3. Expression levels of up to 490 mg/kg of recombinant mAbs were obtained in *Nicotiana benthamiana* glycosylation mutants by using a viral based transient expression system. The plant-made E559 and 62-71-3, carrying human-type fucose-free N-glycans, assembled properly and were structurally sound as determined by mass spectrometry and calorimetric density measurements. Both mAbs efficiently neutralised diverse rabies virus variants *in vitro*. Importantly, E559 and 62-71-3 exhibited enhanced protection against rabies virus compared to human RIG in a hamster model post-exposure challenge trial. Collectively, our results provide the basis for the development of a multi-mAb based alternative to RIG.

## Introduction

Rabies is a zoonotic viral disease that continues to have no effective treatment after onset of symptoms. Typically, infection occurs after a bite from an infected animal, principally the domestic dog. Other animal species, notably wild carnivores and bats, serve as reservoirs of the rabies virus. Post exposure prophylaxis (PEP) is highly effective when administered promptly. The recommended PEP regimen includes immediate administration of Rabies Immune Globulin (or RIG) from pooled sera taken from hyper-immunized horses (ERIG) or humans (HRIG), as well as vaccination with inactivated Rabies Vaccine and thorough wound cleansing [1, 2].

The majority of the approximately 55,000–70,000 annual human rabies fatalities occur in the developing world, yet access to RIG for adequate PEP is still poor in those countries due to affordability and availability [3, 4]. Both HRIG and ERIG are often in short supply due to the exponential increase in demand for PEP in recent years. In addition, RIG suffers shortcomings such as inconsistency between batches, potential for contamination with blood-borne diseases and, in particular for ERIG, occasional adverse allergic reactions such as serum sickness or anaphylactic shock are observed [5]. For these reasons, an international drive to develop alternative PEP biologics, led by the World Health Organisation (WHO), is underway and replacement of RIG with a safer, efficacious and potentially more economical alternative biologic remains a priority. With the involvement of the WHO Collaborating Centres for Rabies Surveillance and Research, several mouse-derived monoclonal antibodies (mAbs) with rabies virus neutralizing activity have been identified [6]. These mAbs have been targeted for future development to replace RIG as components of a safer new generation for PEP that is affordable for cost-sensitive markets.

Clearly, mAbs have several advantages over RIG including better consistency, improved safety, and with humanization, improved tolerance in patients [7, 8]. Alternatively, due to the specificity of individual neutralizing mAbs for different epitopes on the rabies virus glycoprotein, mAb-based products may have limited potential unless they are formulated as a cocktail to avoid virus escape, improve potency and to broaden their viral neutralization breadth, because there is no single pan-reactive mAb against such diverse lyssaviruses documented to date [9]. Given the specificity of mAb 62-71-3 for antigenic site I, all proposed cocktails from the WHO program so far are envisaged to include 62-71-3, and one of mAbs E559.9.14, M727-5-1, M777-16-3 or 1112–1. Among these, mAb E559 has a broad rabies virus isolate breadth of specificity and potency [6, 9].

The current study describes the plant-based recombinant expression, purification, structural and functional characterisation (in vitro and in vivo) of humanized anti rabies mAbs E559 and 62-71-3. mAbs were expressed in ∆XT/FT, a *Nicotiana benthamiana* mutant supporting the synthesis of glycan-optimized fucose-free mAbs [10]. Transient expression in plants using virus based vectors was selected as a highly scalable, rapid production alternative to mammalian cell (e.g., CHO cell) culture-based production. Plant expressed mAbs efficiently neutralized a set of virus strains in a cell based Rapid Fluorescent Focus Inhibition test (RFFIT) assay. Moreover, mAbs exhibited enhanced in vivo potency compared to HRIG as determined by a hamster viral challenge model.

## Results/Discussion

### Recombinant Expression of full length chimeric IgG mAb E559 and 62-71-3 in *Nicotiana benthamiana*

Variable domains from light and heavy chain (V_H_ and V_L_) from murine mAbs E559 and 62-71-3 were fused to the constant domain (C_H_ and C_L_) from human IgG_1_. These chimeric constructs were plant codon-optimised light chain (LC) and heavy chain (HC) expression constructs with two different signal peptides. These chimeric Ab genes were cloned into two non-competing plant viral vectors, tobacco mosaic virus (TMV) and potato virus X (PVX) backbones [11]. The LC and HC vectors were combined and infiltrated into glycoengineered ΔXTFT Nicotiana benthamiana plants [10] that were monitored for expression of assembled IgG. For both mAbs E559 and 62-71-3 the highest expression levels (456 mg/kg and 455 mg/kg respectively) determined by ELISA were attained during initial expression evaluation using the murine signal peptide in the TMV-HC and the PVX-LC combination (Table 1). The expression levels remained constant upon upscaling the procedure in a highly regulated contract manufacturer environment. These expression levels are higher than that observed when antibodies were expressed using transgenic approaches [12, 13]. These data provide a suitable basis for modelling a scaled-up, economically viable manufacturing process.

**Table 1:** MAb E559 and 62-71-3 expression levels obtained using various combinations of either PVX or TMV-based expression vectors with either the murine (m) or rice alpha amylase (r) signal peptide.

Molecule	Vector combinations	Green tissue (g)	Expression (μg/g)
E559	TMV-rHC +PVX-rLC	10	17
E559	TMV-mHC + PVX-mLC	10	456
E559	PVX-rHC + TMV-rLC	10	67
E559	PVX-mHC + TMV-mLC	10	87
62-71-3	TMV-rHC + PVX-rLC	10	339
62-71-3	TMV-mHC + PVX-mLC	10	455
62-71-3	PVX-rHC + TMV-rLC	10	106
62-71-3	TMV-mHC + PVX-mLC	10	278
E559	TMV-mHC + PVX-mLC	1000	349
62-71-3	TMV-mHC + PVX-mLC	1000	493

### Analytical characterisation of plant-produced chimeric mAbs E559 and 62-71-3

Most mAbs can be subject to many potential modifications, including proteolytic clipping, glycosylation, deamidation and oxidation, all of which can affect their efficacy and formulation stability [14]. Therefore, it is important to comprehensively characterise biochemical properties and molecular structures.

**Fig 1:**
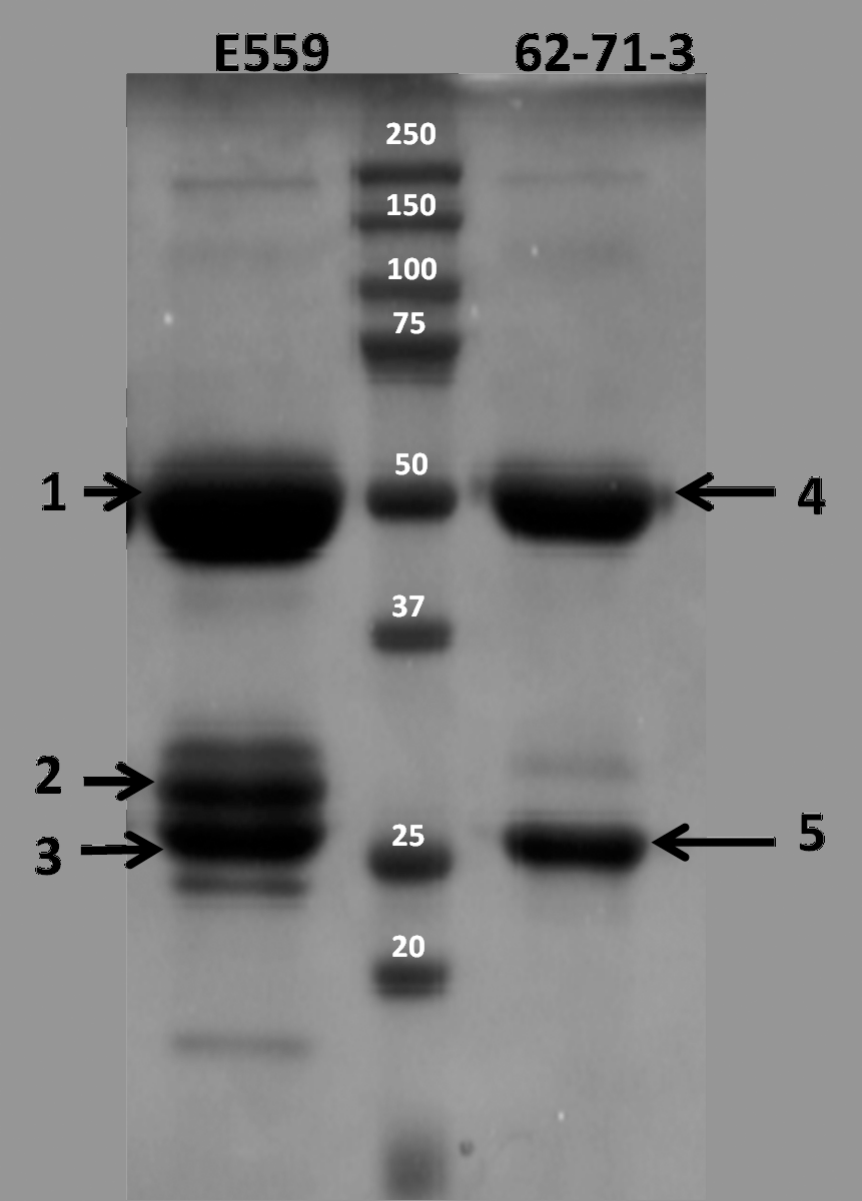
SDS-PAGE analysis of purified E559 and 62-71-3 mAbs. The middle lane was a PageRuler Prestained Protein Ladder, with the sizes indicated in kDa. The numbered E559 and 62-71-3 bands (1–5) were used for further analysis.

**Fig 2:**
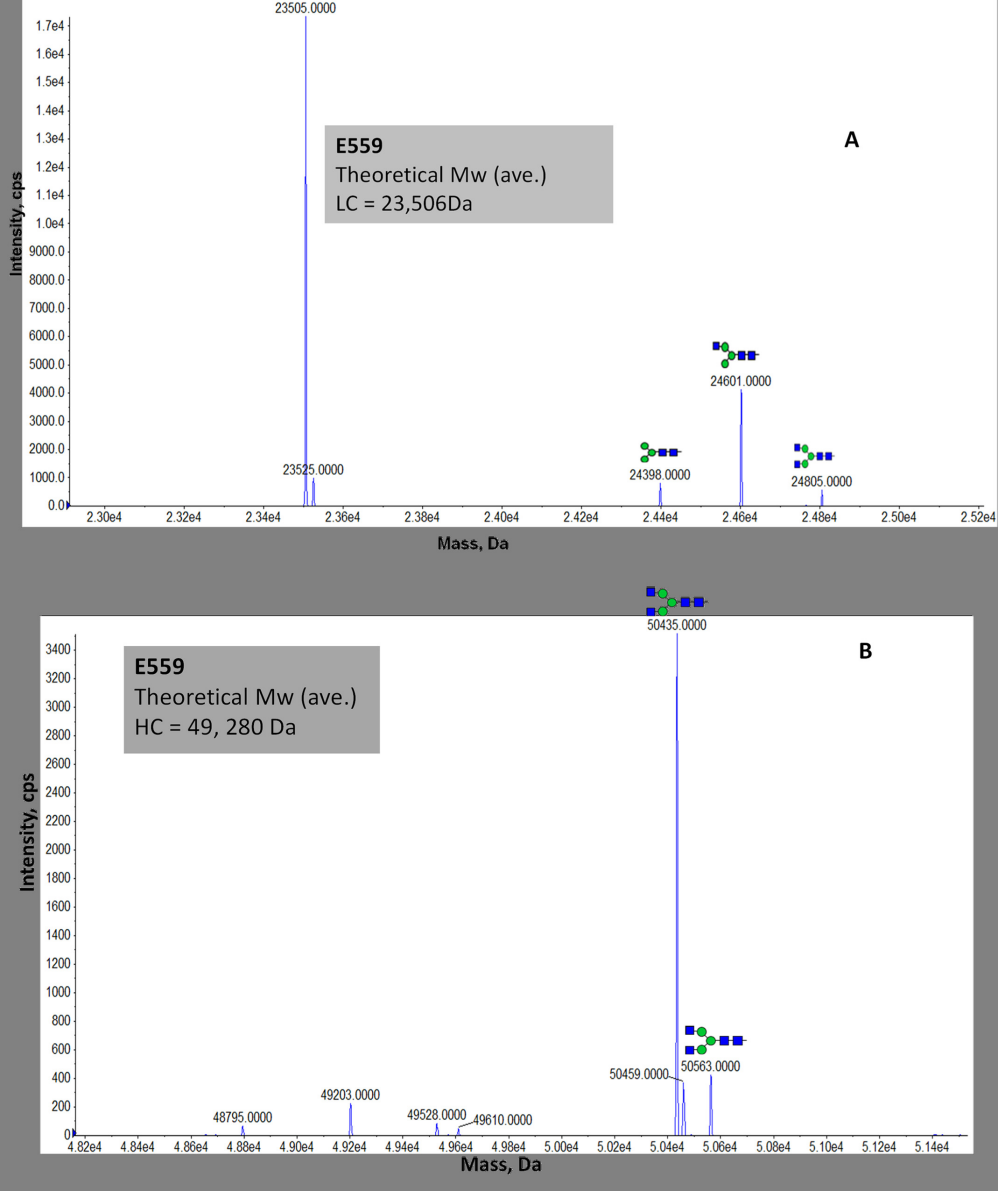
**Deconvoluted spectrum of intact, reduced E559 LC (A) and intact, reduced E559 HC (B)**.Theoretical molecular weights for LC and HC indicated. Detected N-linked glycoforms are shown. The N-glycan nomenclature used was from www.proglycan.com.

Protein A-purified mAbs were characterized by SDS-PAGE under reduced conditions (Fig 1). Both E559 and 62-71-3 heavy chain (HC) bands migrated to their predicted MWs (50 and 25 kDa). Using online LC-ESI-TOF MS was established to elucidate the identity of the mAbs. The deconvoluted multiply charged spectrum of reduced E559 LC is shown in Fig 2(A). The major peak in the spectrum matched the theoretical LC MW of 23,505.87Da. The peaks at 24,398Da, 24,601Da and 24,805Da matched the water-eliminated complex glycans GlcNac_2_Man_3_, GlcNac_2_Man_3_GlcNac_1_ and GlcNac_2_Man_3_GlcNac_2_, respectively. In the HC region of E559 only the glycosylated species were observed whilst the native peak at 49,280.18Da was below the limit of detection (Fig 2B).

**Fig 3:**
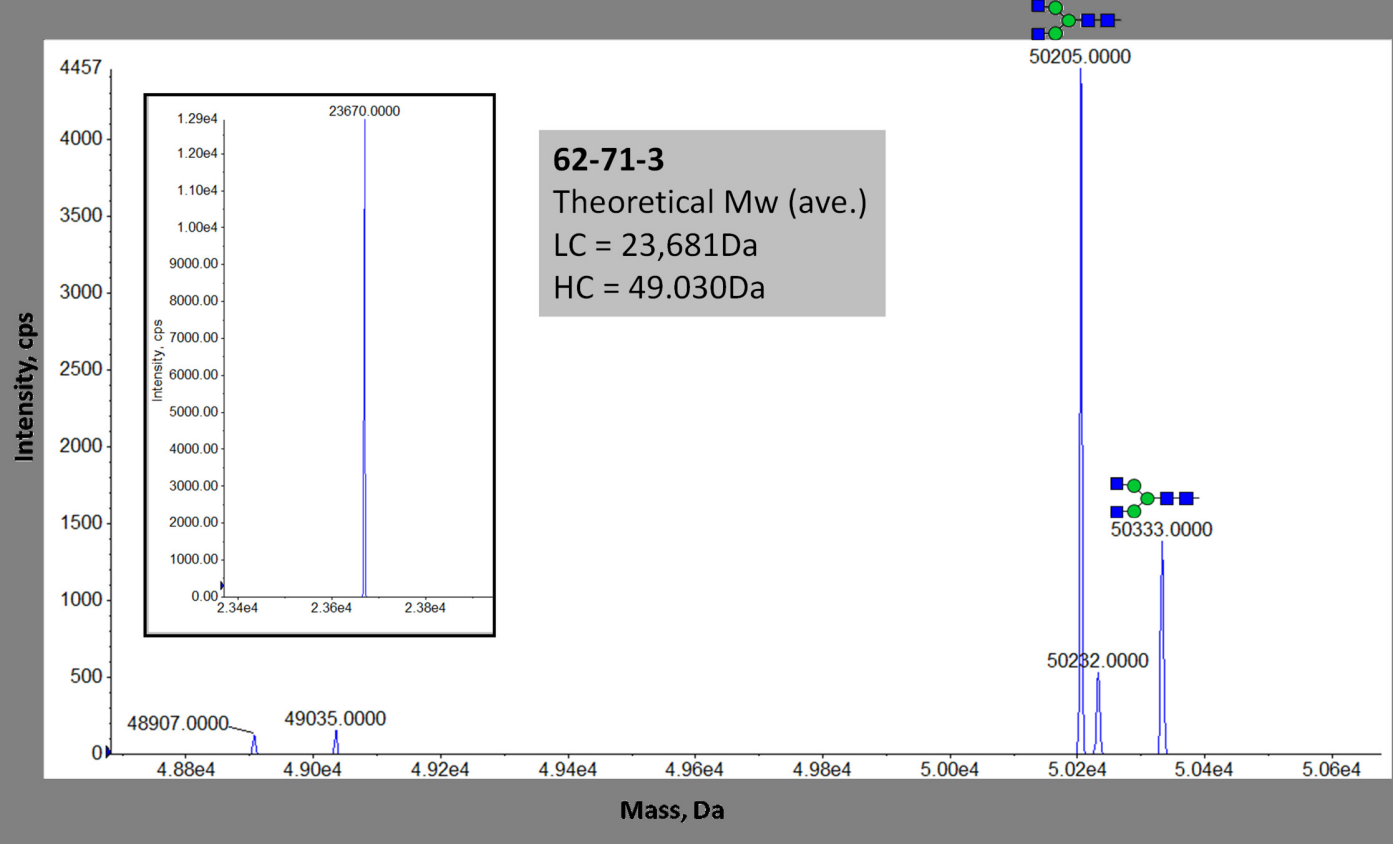
Deconvoluted spectrum of intact, reduced 62-71-3 HC. The inset shows the zoomed-in LC region with theoretical molecular weights for LC and HC indicated. Detected N-linked glycoforms are shown. The N-glycan nomenclature used was from www.proglycan.com.

The deconvoluted multiply charged mass spectrum of reduced 62-71-3 indicated one major peak in the region of the light chain (LC) at 23,670 Da (Fig 3, inset) and no peaks indicative of glycosylation. A putative glycosylation peak was detected at 50,333 Da, 1,298 Da from the 62-71-3 HC of 49,035Da (Fig 3). The mass difference of 1,298Da was indicative of the complex sugar GlcNac_2_Man_3_GlcNac_2_, with a single water molecule eliminated.

Several mAbs, whose N-glycans lack fucose, have been demonstrated to enhance *in vivo* efficacy in different models of viral infection [15–17] due to increased ADCC activity. In addition, afucosylated therapeutic anti-cancer antibodies can exhibit superior in vitro and in vivo efficacy [18, 19]. For these reasons, the anti-rabies mAbs were expressed in the ΔXTFT *Nicotiana benthamiana* host, which is a glycosylation mutant synthesizing predominantly fucose free GnGn glycan structures [10].

**Fig 4:**
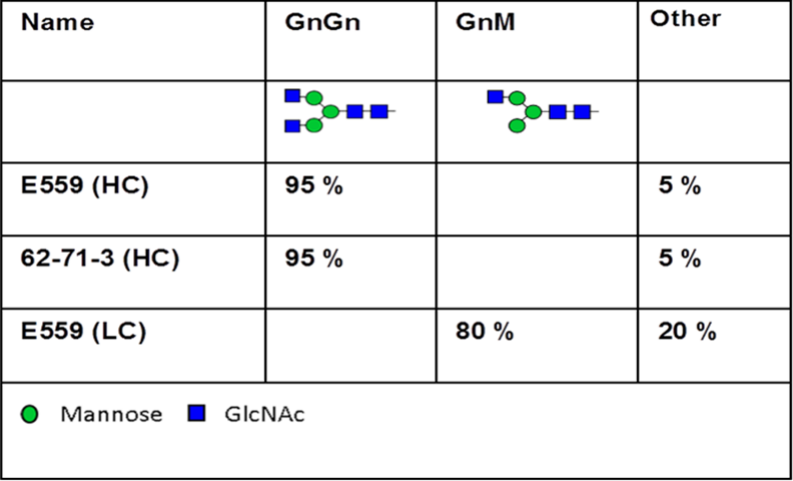
N-linked glycans on the anti-rabies mAbs. N-glycosylation profile from E559 HC and LC and from 62-71-3 HC as determined by LC-ESI-MS of glycopeptides obtained upon trypsin digestion. Numbers represent the presence of the different glyco-species in percent of total glycan. The N-glycan nomenclature used was from www.proglycan.com.

To elucidate site-specific glycosylation of the antibodies, respective glycopeptides of HC and LC were analysed by LC-ESI-MS after protein tryptic digest [20]. The glycopeptide profiles of 62-71-3 and E559 HCs were identical and exhibited a single dominant N-glycan species i.e. GlcNac_2_Man_3_GlcNac_2_ (referred to as GnGn) (Fig 4). The major glycan species on the LC of E559 refers to GlcNac_2_Man_3_GlcNac_1_ (GnM). As expected, the LC of 62-71-3 yielded no glycopeptides (data not shown) corroborating findings from the intact LCMS analyses (Fig 3). Our data show that the molecular weight of each mAb precisely matched the mass expected from the deduced amino acid sequence and had the expected glycan profile.

### Secondary and Tertiary structural characterization

To determine structural integrity of plant produced E559 and 62-71-3 secondary and tertiary structure of E559 62-71-3 were determined using Circular Dichroism Spectroscopy (CD: secondary structure probe) and Fluorescence Spectroscopy (FS: tertiary structure probe).

**Fig 5:**
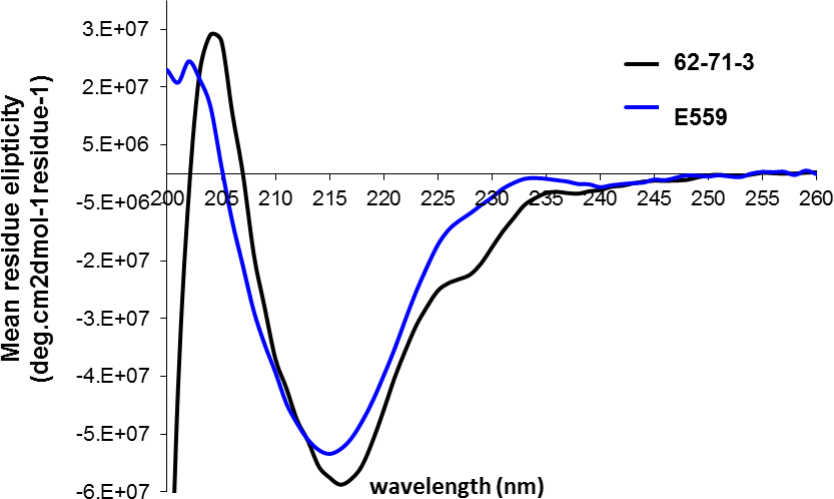
Far-UV CD spectra of humanised 62-71-3 (black) and E559 (blue) mAbs.

**Fig 6:**
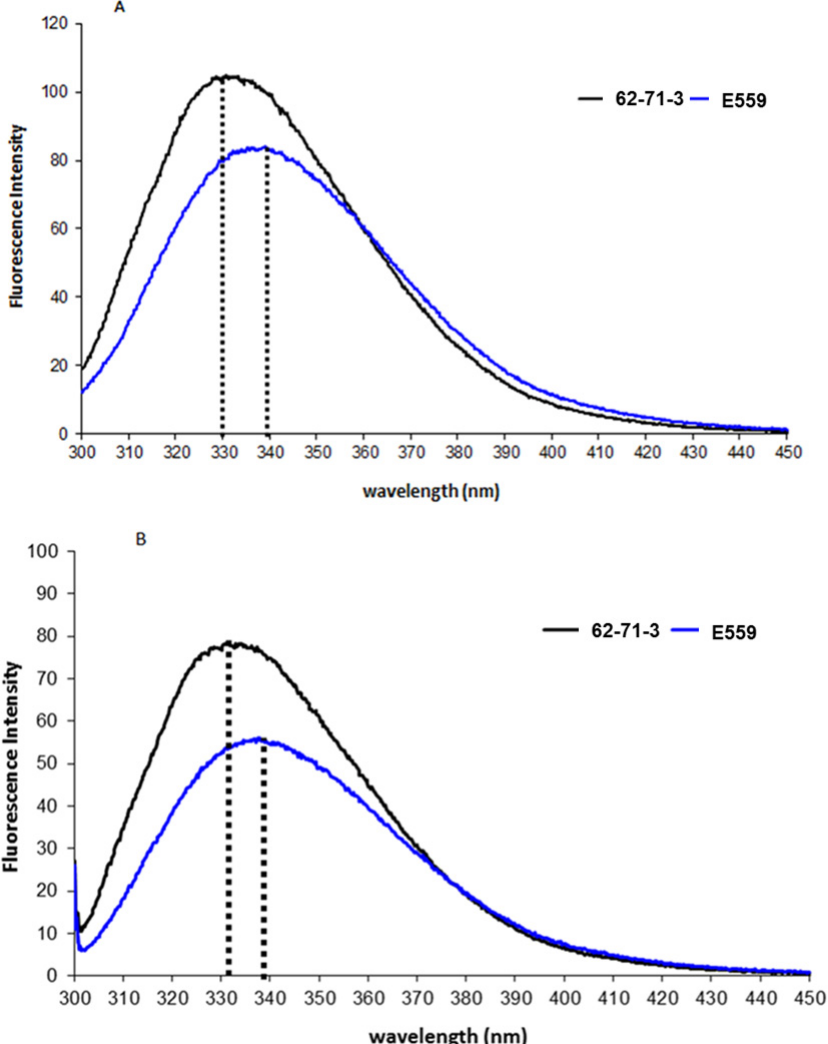
Fluorescence emission spectra of 62-71-3 (black) and E559 (blue). The mAbs were excited at 280 nm (A) and 295 nm (B). λ_emm max_ for each mAb is marked with a dotted line.

The spectra of native mAb molecules were expected to be dominated by β-sheets with few α-helix conformations found in typical antibodies [21]. The Far-UV CD spectra of the two mAbs were closely related with both mAbs exhibiting minima in the 217 nM region indicating that the secondary structural content is indeed dominated by β-sheets (Fig 5). The tertiary and quaternary structures of E559 and 62-71-3 were compared using FS. Both excitation at 280 nm (combined excitation of Trp and Tyr residues) and 295 nm (selective excitation of Trp residues) were used to monitor global differences between the two mAbs. The E559 has 19 Tyr and 10 Trp residues in the HC and 10 Trp and 2 Tyr in the LC. The 62-71-3 mAb has 18 Tyr and 9 Trp residues in the HC and in the LC it has 10 Trp and 2 Tyr, with residues distributed in a similar manner. At both excitation wavelengths, the λ_emm max_ of E559 was shifted to longer wavelengths. This observation indicated a more exposed environment of Trp and Tyr residues for E559 suggesting a more loosely packed quaternary conformation as compared to 62-71-3 (Fig 6).

### Thermal stability

**Fig 7:**
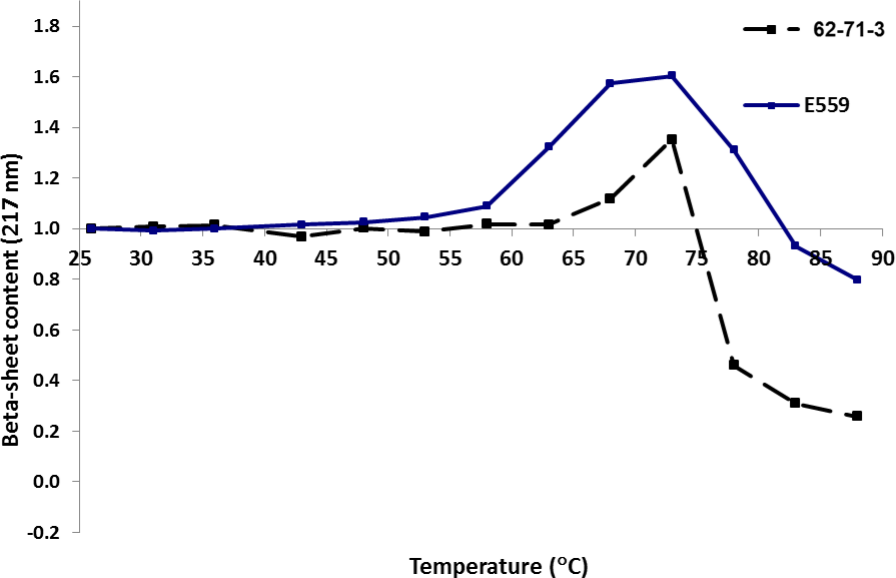
Changes in the β-sheets content of 62-71-3 (black) and E559 (blue) mAbs with increasing temperature.

To determine which mAb is more vulnerable to heat induced degradation, the thermal stability was measured by monitoring changes in secondary structural content [22]. The samples were heated continuously at 5°C per minute from 25 oC to 90 oC and far-UV spectra were measured in the region 180–260 nm. Since both mAb structures are dominated by β-sheets, changes at the 217 nm minima, indicative of β-sheet content, was monitored [21]. Differences were observed from 50–55 oC indicating possible rearrangement in secondary structural content in the case of E559. On the other hand, changes in β-sheet content, for 62-71-3, were only observed above 65 oC (Fig 7). Treatment with antibody combinations can be challenging because in spite of their common structure, individual mAbs often have unique and unpredictable responses to their environment related to their stability [23]. This will also be the case with the envisaged E559 / 62-71-3 cocktail where the data suggest E559 is less thermostable than 62-71-3.

### Neutralisation potency of plant-made E559 and 62-71-3

The two murine mAbs E559 and 62-71-3 were reported to recognise two complementary sites on the rabies virus glycoprotein [6, 9]. In the current study, it was important to confirm that the chimeric, plant-made versions were effective and secondly, to determine their complimentary efficacy or neutralisation pattern against rabies virus isolates in the context of their potential future application as a cocktail rabies PEP biopharmaceutical.

**Fig 8:**
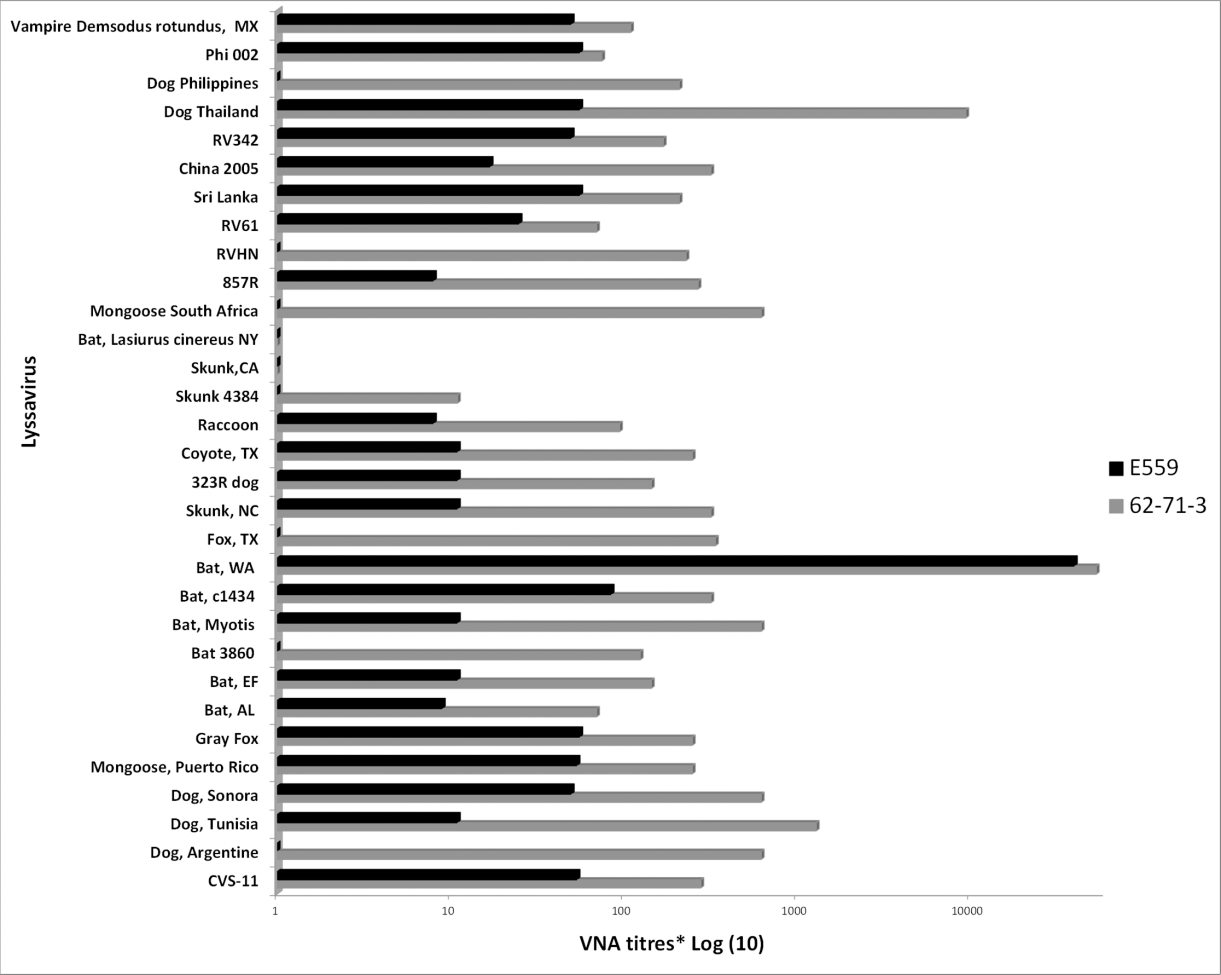
Graphic depiction of *in vitro* Rabies neutralisation activity of mAbs E559 and 62-71-3.

To test the breadth and coverage of the plant-made E559 and 62-71-3 on 31 laboratory and field isolates of rabies virus, a modified Rapid Fluorescent Focus Inhibition test (RFFIT) was conducted and the neutralisation activity was determined as a 50% end point neutralisation (reciprocal titre). Table 2 and Fig 8 show the neutralisation activities. As expected the mAbs neutralised the laboratory strain CVS-11 but had varied neutralisation activity levels on field isolates of rabies virus from different parts of the world. MAb E559 neutralised all the isolates except Bat 3860, Fox (TX), Dog (Philipine and Argentine), RVHN and Mongoose (South Africa), while both mAbs were not active against Skunk (CA) and Bat *Lasiurus cinereus* (NY). Because of the difficulty of transferring isolates across national borders, the sample viruses had only two isolates from African countries. The Dog (Tunisia) isolate was neutralised by both mAbs, while mAb 62–713 neutralised the Mongoose (South Africa) isolate while mAb E559 could not. Antibody E559 binds to the discontinuous antigenic sites II while 62-71-3 binds to antigenic site I of the Rabies virus glycoprotein (RVG). As these antibodies have different binding sites, they provide the capacity to simultaneously bind RVG. Use of these two Abs in a cocktail will enhance the neutralization of wider spectra of rabies viruses and reduce the chances of virus escape [9].

**Table 2:** Fifty percent end-point neutralisation activity (reciprocal titre) of E559 and 62-71-3 in a modified Rapid Fluorescent Focus Inhibition Test (RFFIT).

*Rabies Virus*	VNA titres		*Rabies Virus*	VNA titres	
	62-71-3	E559		62-71-3	E559
CVS-11	280	54	Raccoon	95	8
Dog, Argentina	625	<5	Skunk, 4384	11	<5
Dog, Tunisia	1 300	11	Skunk, CA	<5	<5
Dog, Sonora	625	50	Bat, *Lasiurus cinereus* NY	<5	<5
Mongoose, Puerto Rico	250	54	Mongoose, South Africa	625	<5
Gray Fox	250	56	857R	270	8
Bat, AL	70	9	RVHN	230	<5
Bat, EF	145	11	RV61	70	25
Bat, 3860	125	<5	Sri Lanka	210	56
Bat, *Myotis*	625	11	China 2005	320	17
Bat, c1434	320	85	RV342	170	50
Bat, WA	53 887	40 269	Dog, Thailand	9 500	56
Gray Fox, TX	340	<5	Dog, Philippines	210	<5
Skunk, NC	320	11	Phi 002	75	56
Dog, 323R	145	11	Vampire *Demsodus rotundus*, MX	110	50
Coyote, TX	250	11			

Titres < 5 did not neutralise at the concentration tested (1 mg/ml of each mAb).

### In vivo efficacy

**Fig 9:**
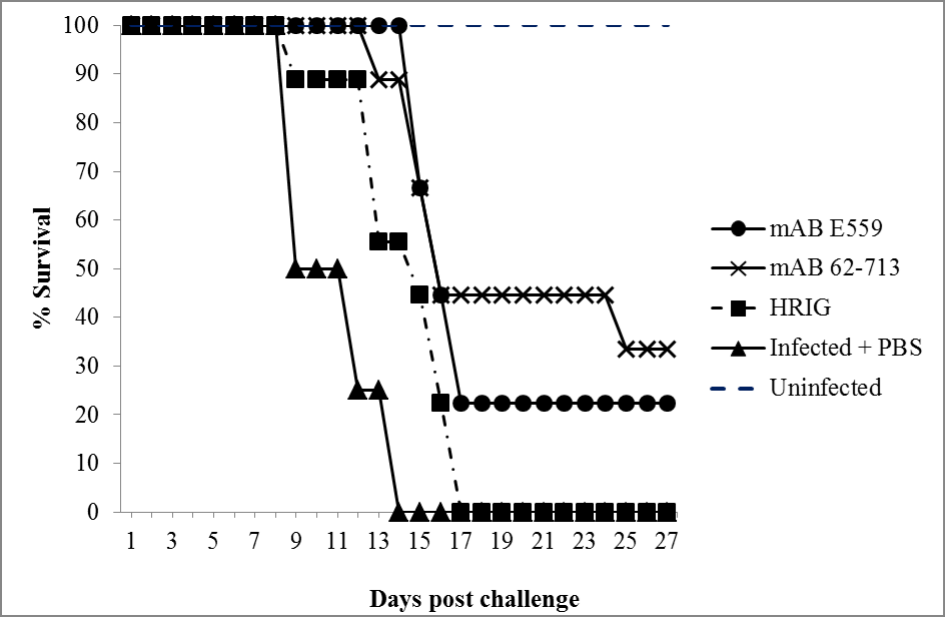
Hamster survival curve after infection with rabies virus and administration of PBS, RIG or mAbs. For infected and treated groups, 9 hamsters were in each group, while untreated control groups had 4 hamsters.

The *in vivo* potency of the plant-produced mAbs was tested in a challenge experiment with female Syrian hamsters infected with CVS-11 (Fig 9). The infected control group did not survive beyond 14 dpi confirming the lethality of the viral inoculum. Animals receiving treatment were administered 2 International Units (IU) of mAb or HRIG. Animals treated with the plant-made mAb E559 (Group 2) showed 100% protection at 14 dpi, slightly higher than that observed with mAb 62-71-3 (Group 3), where 86% of the animals survived at the same time point. At 28 dpi, 33% of animals treated with mAb 62-71-3 and 20% of animals treated with E559 survived; while all animals treated with HRIG died. Collectively, the results show the following order of efficacy: mAb mAb62-71-3>E559>HRIG. The results suggest that the efficacy of plant-made candidates for the cocktail (mAb E559 and mAb 62-71-3) could surpass the commercially available HRIG.

### Hurdles to further development and the way forward

Although rabies is fatal after the onset of clinical signs, the disease can be readily prevented with existing options for PEP. The compelling need to find a less expensive and safe alternative for RIG alone is not sufficient to pave a smooth path for further development of recombinant mAb-based PEP for implementation in the clinic. The biggest barrier to reaching the market is the complicated and untried clinical development path for the replacement of efficacious immunotherapies for lethal but neglected infectious diseases, such as rabies. In most countries, human efficacy trials for replacement products for these diseases are impossible due to ethical considerations. The US FDA has since 2002 made available an alternative ‘animal rule’ pathway for development where efficacy is established in well-controlled model animal trials and safety in normal human trial pathways. The situation in other parts of world is more complicated and will require coordinated efforts for encouraging regulatory reform on a global level.

There is no set clinical or regulatory path for this type of prophylaxis where it is needed the most, irrespective of whether it was produced from the somewhat novel plant based platform or from the more established mammalian cell culture systems (e.g. CHO cells). Proactive and early engagement with Asian and African regulatory authorities, such as South Africa’s Medicines Control Council, is underway to sensitise them on approval frameworks and market entry elsewhere in the world.

In addition to ethical and regulatory hurdles, global plant-based manufacturing capacity for clinical grade biologics is currently a major barrier. The need for cGMP accredited facilities to enable at-site production for trials and subsequent market penetration has been highlighted before [24]. A shared regional facility model is currently being considered in South Africa, but the main limitation is poor availability of funding from already overstretched public sector budgets.

## Materials and Methods

### Engineering, cloning and expression of mAbs E559 and 62-71-3

*Nicotiana benthamiana* codon optimised genes of mAbs 62-71-3 and E559 light and heavy chains were synthesized (Geneart) on the basis of available sequence information. Variable region gene sequences (V_H_ and V_L_) from murine mAbs E559 and 62-71-3 were grafted onto constant region (C_H_ and C_L_) sequences from human IgG1. These complete chimeric LC and HC gene sequences were then cloned into two different vectors (ICON Genetics MagnICON vectors pICH26211 and pICH31160 with TMV and PVX viral backbones [11] respectively) with rice alpha amylase and murine signal peptide sequences (designated r and m). This resulted in a total combination of 16 vectors.

*Agrobacterium tumefaciens* strain ICF320 (ICON genetics, Germany) cultures transformed individually with these vectors were grown and diluted in infiltration medium. To test the expression for each mAb, the 16 gene/vector backbone constructs were infiltrated pair wise in the following combinations: TMV-rHC +PVX-rLC, TMV-mHC + PVX-mLC, PVX-rHC + TMV-rLC, PVX-mHC + TMV-mLC, TMV-rHC + PVX-rLC, TMV-mHC + PVX-mLC, PVX-rHC + TMV-rLC, TMV-mHC + PVX-mLC, TMV-mHC + PVX-mLC, TMV-mHC + PVX-mLC (Table 1). These combinations of vectors were used to transfect 30 days post sow (dps) mutant ΔXTFT glycosylation *Nicotiana benthamiana* plants. Transfection was done by infiltration in a vacuum chamber at 10 mm Hg. Total protein was extracted from 10 g of leaves 7 days post infiltration (dpi), and assayed for antibody titre using ELISA according to standard techniques.

### Production, extraction and purification of antibodies

*N*. *benthamiana* plants (ΔXTFT glycosylation mutants) grown for 30 days were vacuum infiltrated with *Agrobaterium* ICF320 transformed with the optimal combination of LC and HC constructs, as determined above. The recombinant *Agrobacterium* was diluted in infiltration medium before transfection under vacuum at 10 mm Hg. *Nicotiana* plants used were previously reported in [10]. Infiltrated plants were allowed to recover and left in the growth room for transient expression and assembly of antibodies. Plants were harvested 7 dpi and homogenised in extraction buffer containing 100 mM Glycine, 40mM Ascorbic Acid, 1mM EDTA (pH 9.5). A 1:1 buffer (l) to harvested plants (kg) ratio was used. The resulting green juice was clarified by filtration through four layers of cheese cloth followed by centrifugation at 10000 x g for 20 minutes. Clarified green juice was loaded onto equilibrated MAb SELECT SURE Protein A affinity resin (GE Healthcare) for capture and first step purification. After a 5 column volume (CV) wash with buffer containing 50 mM Tris, pH 7.4, bound protein was eluted with pH 3 buffer containing 100mM Acetic Acid and immediately neutralized. Eluted samples were then loaded onto an equilibrated Capto Q column (GE Healthcare) and the flowthrough/wash fraction collected. This mAb-containing fraction was finally polished with Ceramic hydroxyapatite (CHT) chromatography with type II resin (Biorad).

### Analytical characterisation

Purified mAbs 62-71-3 and E559 were reduced using 20 mM DTT for 15 minute at 50°C. Next, approximately 20 pmol total protein was loaded, using 10% ACN/0.1% FA, on a Jupiter C4 reverse phase column coupled via a switch valve to a QSTAR Elite TOF MS equipped with a TurboIon ESI source. Samples were desalted for 5 minute and eluted using a linear ACN gradient (20–50% in 20 minute at 150 ml/min). Charge state envelopes were collected in the range 700–2000 Da followed by deconvolution of multiply charged data via the Bayesian Protein Reconstruct tool of Analyst QS 2.0. The resulting intact masses of the 62-71-3 and E559 mAb light chain (LC) and heavy chain (HC) were compared to theoretical molecular weights (Mws) obtained using the amino acid sequences of each mAb.

The N-glycosylation profile was determined by LC-ESI-MS as previously described by [20]. In brief, purified IgG was separated by reducing SDS-PAGE, Coomassie stained and the heavy light chain band was excised from the gel. Upon S-alkylation and tryptic or tryptic/GluC digestion, fragments were eluted from the gel with 50% acetonitrile and separated on a Reversed Phase Column (150 × 0.32 mm BioBasic-18, Thermo Scientific) with a gradient of 1–80% acetonitrile. Glycopeptides were analyzed with a Q-TOF Ultima Global mass spectrometer (Waters). Spectra were summed and deconvoluted for identification of glycoforms. Glycans were annotated according to the proglycan nomenclature (www.proglycan.com).

Fluorescence emission spectra were recorded using 3 μM E559 and 62-71-3 in the range 280–450 nm. The excitation and emission slit widths were kept at 5 nm. The spectra were recorded at 23°C, buffer corrected and was an average of three accumulations at a scan speed of 200 nm/min. Readings were taken in a quartz cuvette with a 1 mm path-length using a Shimadzu luminescence spectrometer RF-5301-PC v 2.04 software.

Far-UV-CD spectra (190–250 nm) were recorded using 3 μM E559 and 62-71-3. All CD spectra were recorded at 23°C and represent an average of 3 accumulations, at a scan speed of 100 nm/min. The bandwidth used was 1 nm and the data pitch 0.2 nm. All readings were recorded in a 2 mm cuvette using an Applied Photophysics Chirascan spectropolarimeter and the Spectra Manager software v1.5.00. All spectra were buffer corrected. The spectra were normalised by calculating the mean residue ellipticity [θ] deg.cm^2^dmol^-1^residue^-1^ using the equation [θ] = (100xθ)/cn. Where (θ) is the ellipticity signal in mdeg, c (mM) is the protein concentration, n is the number of residues in the protein chain and *l* is the path length in cm. All CD spectra were processed using Pro-Data Viewer v4.1.1.

The thermostability of E559 and 62-71-3 were compared by measuring changes in secondary structure using Circular Dichroism. Ellipticity change at 217 nm, reporting on α- β-sheet regions, were recorded with increasing temperatures in the range 25–90°C. Temperature was increased in 5°C increments with a 5 min equilibration time at each temperature. 3 μM of each mAb was used for the experiment. All CD spectra represent an average of 3 accumulations, at a scan speed of 100 nm/min. The bandwidth used was 1 nm and the data pitch 0.2 nm. All readings were recorded in a 2 mm cuvette using a Applied Photophysics Chirascan spectropolarimeter and the Chirascan software v1.5.00.

### In vitro efficacy

Neutralisation ability of plant-produced mAb E559 and 62-71-3 was tested against a diverse panel of 31 different rabies virus isolates. To determine the virus neutralising antibody (VNA) titres of the two mAbs, a modification of the Rapid Fluorescent Focus Inhibition test (RFFIT) was used. The titres were calculated using 50% end-point neutralisation (reciprocal titre) of E559 and 62-71-3. Titres <5 indicate absence of neutralization at the concentration tested. Each mAb was at a concentration of 1 mg/ml, as determined spectrophotometrically by absorbance at 280 nm wavelength. To summarise the methodology briefly, all virus isolates were adjusted to ~10^4^ ffu/ ml, mixed with an equal volume of antibody dilution, incubated for 1 hour at 37°C followed by addition of Baby Hamster Kidney (BHK) cells. The virus dose was checked by back titration on every test and results were rejected if virus dose was outside pre-determined limits (i.e. mean±2sd for positive serum and 30-100TCID50/ml for challenge virus).

### Animal model challenge

*In vivo* protective activity of the plant-produced mAbs *in vivo* was determined by infecting Syrian hamsters with rabies virus strain CVS-11 in the gastrocnemius muscle followed by administration of the test antibodies as part of a PEP regime. The study design included five groups of six-week-old female Syrian hamsters.

Briefly, 2 IU of the relevant mAb or undiluted HRIG [Rabigam, 150 iu/ml, National Bioproducts Institute, Pinetown, South Africa] were introduced intra-peritoneally into hamsters distributed into four groups of 9 hamsters for the experimental groups and four hamsters each for the control groups. In the mAb control groups, hamsters were injected with phosphate buffer (PBS) only (negative control), or commercial HRIG (Rabigam, obtained from National Bioproducts Institute, Durban, South Africa). The PEP was administered 24 hrs after challenge. In the experimental groups, 50 μl of 1 x 10^6^ TCID_50_/ml of challenge virus standard (CVS-11) was introduced as described in Table 3 below.

**Table 3:** Rabies virus challenge and administration schedule of anti-rabies mAbs in Syrian hamsters.

Group number	Number of hamsters	Treatment	Day 1	Day 2
Group 1	9	Infected & treated with mAb E559	Challenge with CVS	mAb E559
Group 2	9	Infected & treated with mAb 62-71-3	Challenge with CVS	mAb 62-71-3
Group 3	9	Infected & treated with HRIG	Challenge with CVS	HRIG
Group 4	4	Infected & untreated control (PBS only)	Challenge with CVS	PBS
Group 5	4	uninfected & untreated control (PBS only)	None	PBS

### Ethics statement

The animal experimental protocols, animal caging and care as well as end point for the animal experiments were approved by the Agricultural Research Council-Onderstepoort Veterinary Institute (ARC-OVI, South Africa) Animal Ethics Committee for the use of living vertebrates for research, diagnostic procedures and product development. The approval application number was AEC36,09 for project number 15/4 P001. Hamsters that survived for 28 days after infection were euthanized with isoflurane.
